# General and Life-Domain Procrastination in Highly Educated Adults in Israel

**DOI:** 10.3389/fpsyg.2018.01173

**Published:** 2018-07-04

**Authors:** Meirav Hen, Marina Goroshit

**Affiliations:** Department of Psychology, Tel-Hai Academic College, Tel Hai, Israel

**Keywords:** adult procrastination, general procrastination, life-domains, life-domain procrastination, highly educated adults

## Abstract

Procrastination is usually perceived as a general behavioral tendency, and was studied mostly in college students in academic settings. Recently there is a growing body of literature to support the study of procrastination in older adults and in different life-domains. Based on these advances in the literature, the present study examined procrastination in 430 highly educated adults in Israel. Findings showed that respondents reported significantly higher procrastination in maintaining health behaviors and spending leisure time rather in other life-domains. Forty percent of participants reported high procrastination in health behaviors, while only 9.5% reported this level of procrastination in parenting and 1% in the general tendency to procrastinate. Further findings suggested that 25% of respondents reported high procrastination in four or more life-domains, and 40%—in one to three life-domains. The general tendency to procrastinate was moderately associated with procrastination in finance, education, and career life-domains and weekly with other life-domains. Fourteen percent of participants reported that procrastination influenced their life the most in health behaviors, 12% in career and education and 11% in romance and family life. These initial findings contribute to the overall perspective of life-domain specificity of procrastination in adults, and emphasize the importance to further study and develop a life-span perspective.

## Introduction

Procrastination is a common behavior in contemporary societies (Ferrari, 2010). It is believed to be a personality characteristic or a behavioral tendency that is associated with a variety of personal and situational determinants. Procrastination is often conceptualized as the voluntary delay of important and necessary tasks, despite knowing that one will be worse off for doing so (Steel and Klingsieck, 2016). This behavior was found to be associated with many personal, cognitive, emotional, and motivational factors that often lead to a somewhat fragmented representation of this behavior (Rebetez et al., 2015). However, possible outcomes of these delays have been associated with many negative consequences, including mental and physical health problems, ability to achieve academic and career goals and financial well-being (Steel, 2007). Over the years, procrastination was studied mostly in young college students attending academic settings, and was suggested to be strongly associated with a self-regulation failure in face of temptations (Steel, 2007). Further, it was suggested to be a mechanism to avoid difficult tasks, academic failure, anxiety provoking situations as well as a short-term mood-regulation strategy, the inability to delay gratification, a time management problem or a “last-minute” thrill experience (Rice et al., 2012; Grunschel et al., 2013, 2016; Sirois and Pychyl, 2013; Dunn, 2014; Malatincová, 2015; Lowinger et al., 2016).

Recently there is a growing body of literature that supports the need to study adult procrastination from a life-span perspective and in specific life-domains (Klingsieck, 2013; Mann, 2016; van Eerde, 2016). It is argued that these studies will increase our understanding of procrastination in general, and in specific life-domains along the life-span. This knowledge is important for the prevention of procrastination and the development of interventions to address procrastination in life-domains were older adults may be negatively affected. Therefore the present research aimed to contribute to this literature by examining adult procrastination in general and in specific life-domains.

### Adult procrastination

The nature of procrastination in older adults was studied from different angels (Ferrari et al., 2007; Tibbett and Ferrari, 2015). Epidemiological studies in English speaking countries, in Germany, Turkey, and six Spanish talking countries indicated that people procrastinate less as they age, when they are educated, married, have a job and reside in countries with higher levels of self-discipline (Ferrari et al., 2005b, 2007, 2009b; Gröpel and Steel, 2008; Steel and Ferrari, 2013; Beutel et al., 2016). Further, it was revealed that procrastination in older adults is associated with emotional variables such as life regrets (Ferrari et al., 2009a), perceived stress, distress (depression, anxiety, fatigue), and reduced life satisfaction across a broad set of life-domains (Beutel et al., 2016). Health related variables such as: lack of energy (Gröpel and Steel, 2008), hypertension and Cardiovascular disease (Sirois, 2015), and low social well-being (Steel, 2010) were indicated as well.

Interestingly, from a life-span perspective, Ferrari et al. (1995) argued that while it seems that procrastination rates decrease with age, these rates actually seem to reflect different forms of procrastination. They claimed that while the academic specific task related form of procrastination decreases, chronic, dispositional delay behavior patterns are presented, reflecting a chronic engagement in task delays as a maladaptive lifestyle (Ferrari et al., 1995). This perspective was somewhat supported by studies that did not find a strong age effect on the prevalence of procrastination (Steel and Ferrari, 2013; Svartdal et al., 2016). Further findings indicated that 50% of adult respondents reported procrastinating at times, while 20% of them claimed to be procrastinating most of the time (Harriott and Ferrari, 1996). Other studies indicated that chronic procrastination in adults was higher in professionals (white-collar employees), in corporate settings (Ferrari et al., 2005a) was associated with unhappy memories (Tibbett and Ferrari, 2015) and revealed different time orientations for non-chronic procrastination (Díaz-Morales et al., 2008).

### Adult procrastination in specific life-domains

Adult procrastination was also studied in specific life-domains (Nguyen et al., 2013). These studies examined procrastination and its' correlates in designated life-domains, trying to better understand the situational antecedents and consequences of this behavior. Bedtime procrastination, for instance, “the needlessly and voluntarily delaying going to bed, despite foreseeably being worse off as a result” (Nauts et al., 2016, p. 80), was studied in the framework of self-regulation and was found to be highly prevalent among Dutch adults. It was negatively correlated to self-regulation and positively to insufficient sleep, and general procrastination (Kroese et al., 2014), and it mediated the relationship between self-regulation and insufficient sleep (Kroese et al., 2016). Another study indicated that bedtime procrastination was also correlated with reported aversive bedtime routines (Nauts et al., 2016).

Procrastination in conducting health behaviors was first studied in college students suggesting a negative association between general procrastination and intentions to engage in health behaviors (Sirois, 2004). Further, it was studied in a community adult sample and found that procrastination in adults was associated with higher stress, more acute health problems, and the practice of fewer wellness behaviors (Sirois, 2007). Procrastinators also reported fewer household safety behaviors, and less frequent dental and medical check-ups (Sirois et al., 2003; Sirois and Tosti, 2012).

As timely performance is a requirement of most jobs, procrastination may be particularly problematic in the workplace (van Eerde, 2016). Malachowski (2005) estimated that the average employee loses 2 h of each work day to such time-wasting activities as surfing the Internet for personal use and socializing with colleagues, resulting in a salary loss of $759 billion per year in the American workforce alone. Studies that explored the antecedents of procrastination in the workplace observed procrastination to be partially related to occupational stressors, including psychological detachment and fatigue (DeArmond et al., 2014), negatively associated with future time orientation, and positively associated with present-fatalistic time orientation (Gupta et al., 2012). Other studies explored job characteristics related to procrastination and suggested that people tend to procrastinate on tasks that are not stimulating or unpleasant or difficult or tasks that are imposed upon them (Blunt and Pychyl, 2005).

### The present study

Following the above literature, and Klingsieck's (2013) initial study on students' procrastination frequencies in six different life-domains the present study aimed at examining general and life-domain procrastination in highly educated adults. Following Ferrari et al. (1995) and in an attempt to contribute to the life-span perspective of procrastination, we studied higher educated adults that were, at one point, college students and possibly engaged in academic procrastination. We studied their overall tendency to procrastinate and their procrastination in 11 different life domains (health, career, education, family, friends, self, leisure-time, community, finance, and parenting). We also asked the participants to reflect in which life-domain procrastination had the most influence on their lives. Accordingly, our research questions were:

1. What are the mean levels of the general procrastination tendency and procrastination in each life-domain in highly educated adults?
2. What are the frequencies of high general procrastination and procrastination in each life-domain?
3. In how many life-domains did the respondents state that they experience high procrastination?
4. What are the relationship between life-domain procrastination and the general tendency to procrastinate?
5. In which life-domains do respondents perceive their procrastination as the most influential on their lives?

## Methods

### Participants and procedure

The sample consisted of 430 Israeli adults in the age range from 26 to 70 (*M* = 43.41, *SD* = 11.13). Seventy percent of the sample was female; approximately 82% stated that they are married and 58% are secular. All participants reported that they are employed and 75% of them are full-time workers. In an attempt to explore life-domain procrastination in highly educated adults, the inclusion criterion required that participants have some college degree. The sample included participants from free professions (such as school teachers, psychologists, engineers, computer programmers, medical doctors and nurses, mean years of education = 18, *SD* = 3), 80% of the sample were residents of urban areas.

Recruitment of participants was conducted by an Israeli online data collection company, which employs a panel of over 100,000 participants representing the total Israeli Jewish population (www.ipanel.co.il). The questionnaire was distributed online using Qualtrics software (www.qualtrics.com). The recipients who received the link to the survey were first directed to a page containing an informed consent letter; they were required to provide their informed consent before proceeding to the survey itself.

### Instruments

We measured life-domain procrastination (*LDP*, see Appendix 1) based on three main sources: Klingsieck's (2013) research on life-domain procrastination, Ferrari et al.'s (2009a) work on life-domain regret and Goroshit et al.'s (2018) paper on life-domain procrastination regret. We asked the participants to what extent they procrastinate (from 1 = *not at all/rarely* to 5 = *very often/all the time*) in eleven life-domains: family, friends, leisure, health, finances, career, education, self, community, parenting, and romance. We presented an example of each life-domain (e.g., “To what extent do you procrastinate in the field of family relations, like calling or meeting your parents or siblings, going to family dinners” or “To what extent do you procrastinate in financial issues, like investments, saving programs, going to your bank?”). We also asked an open-ended question regarding life-domain procrastination: “In which life-domain your procrastination influenced you the most?”

In addition, we measured the general tendency to procrastinate, using the Adult Inventory of Procrastination (*AIP*; McCown and Johnson, 1989), which is a 15-item, 5-point Likert scale (from 1 = *false for me* to 5 = *true for me*) that included items such as “I do not get things done on time” and “I am not good at meeting deadlines.” Previous studies reported that the AIP was a valid and reliable measure of behavioral procrastination with a Cronbach's alpha reliability of 0.79–0.83 and a test-retest reliability score of 0.71 (Ferrari, 1994; Ferrari et al., 1995). For the current study, we calculated a mean score of the AIP items (α = 0.88).

## Results

**Figure 1:**
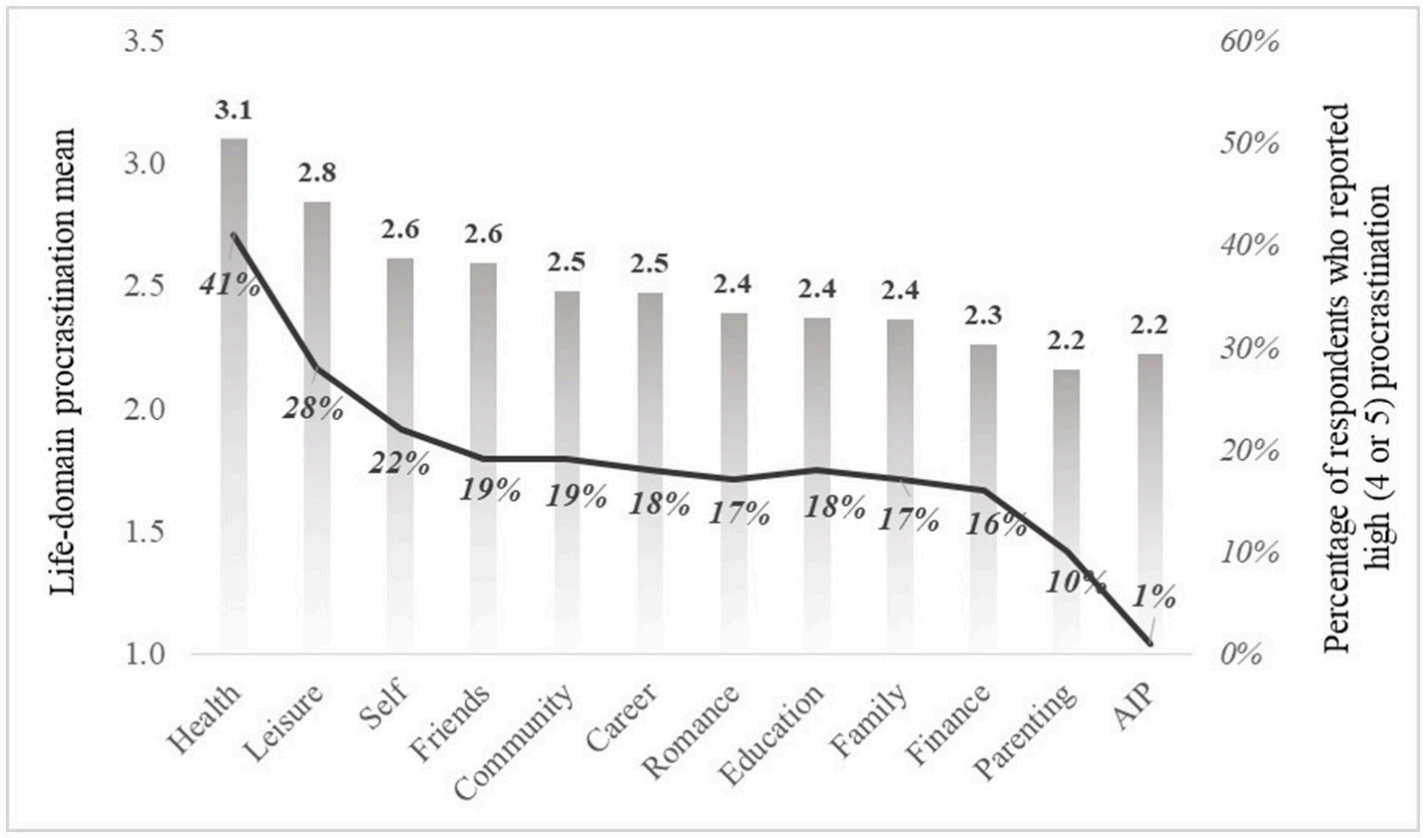
Mean levels of life-domain procrastination (bars) and percentage of respondents who reported high (4 or 5 in scale 1–5) procrastination in each life-domain (line).

To reply our first and second questions regarding the levels of the general tendency to procrastinate and life-domain procrastination in our sample, we looked at the distributions of variables (means, SDs, and frequencies). We also examined the percentage of respondents that reported they procrastinate often (score of 4 in a scale 1–5) or very often (score of 5 in scale 1–5) in every life-domain and in general procrastination. This allowed us to see the frequencies of respondents that can be defined as “high procrastinators” in each life-domain and in general procrastination (see Table 1 and Figure 1).

**Table 1:** Means, standard deviations, and frequencies of procrastination in different life-domains and general tendency to procrastinate (AIP).

**Procrastination in different life-domains**	**M**	**SD**	**% of high procrastinators (score of 4 or 5 in scale 1–5)**
Career	2.47	1.06	17.6
Community	2.48	1.13	19.4
Education	2.37	1.19	18.2
Parenting	2.16	1.02	9.5
Family	2.36	1.13	17.1
Finance	2.26	1.13	15.5
Friends	2.59	1.07	19.3
Health	3.10	1.29	40.7
Leisure	2.84	1.14	27.7
Romance	2.39	1.14	17.2
Self	2.61	1.11	21.9
AIP	2.22	0.67	1

Results indicated that the most frequently procrastinated life-domain was health; leisure and self were at the second and the third place, respectively. The three less frequently procrastinated life-domains were family, finance and parenting. Interestingly, the mean AIP score was similar to the less procrastinated life-domains. We also found that 40.7% of respondents reported high procrastination in maintaining health behaviors, 27.7 % in spending leisure time, 21.9% in self-development.

In order to test whether there are significant differences in procrastination between the life-domains, we ran repeated measures ANOVA and used a *post-hoc* contrasts test to explore the differences. This test showed that there are significant differences between the life-domains [*F*_(10, 2470)_ = 24.33, *p* < 0.001] with effect size of 0.09, and that the procrastination in leisure and health life-domains is significantly higher than in other domains.

To test our third question regarding the number of highly procrastinated life-domains, we calculated in how many domains respondents reported high (4 or 5 on the scale of 1–5) procrastination. We found that 25% of the participants reported high procrastination in four to eleven life-domains, 40%—in one to three domains and 35% of participants reported low procrastination in all life-domains (see Table 2).

**Table 2:** Distribution of number of life-domains (out of 11) reported as highly procrastinated (4 or 5 on the scale of 1–5).

**Number of life-domains**	***N***	**%**	**Cumulative %**
0	156	35.2	35.2
1	72	16.3	51.5
2	61	13.8	65.3
3	44	9.9	75.2
4	50	11.3	86.5
5	29	6.5	93
6	16	3.6	96.6
7	5	1.1	97.7
8	5	1.1	98.8
9	3	0.7	99.5
10	2	0.5	100
Total	443	100	

To answer our fourth question concerning the relationships between life-domain procrastination and the general tendency to procrastinate, we ran Pearson bivariate correlations between life-domain procrastination items and the AIP score. Results showed that life-domains such as career, education and finance share more variance with AIP (correlations around 0.4 to 0.5), rather life-domains such as community, family and health (correlations around 0.3) or parenting, friends and romance (correlation around 0.1 to 0.2). This suggests that the AIP, as a measure of general tendency to procrastinate, is limited in terms of capturing the differences between the tendencies to procrastinate in different life-domains.

To compare between the correlations of AIP with procrastination in each life-domain we used an online calculator developed by Lenhard and Lenhard (2014). These comparisons showed that there were three distinguished groups of correlations in terms of their strengths: the first group was correlations from 0.1 to 0.18 (correlations between AIP and self-, romance, friends, and parenting life-domains), the second group was correlations from 0.25 to 0.29 (correlations between AIP and leisure-time, health, family, and community life-domains) and the third group was correlations from 0.41 to 0.48 (correlations between AIP and career, education and finance life-domains).

**Figure 2:**
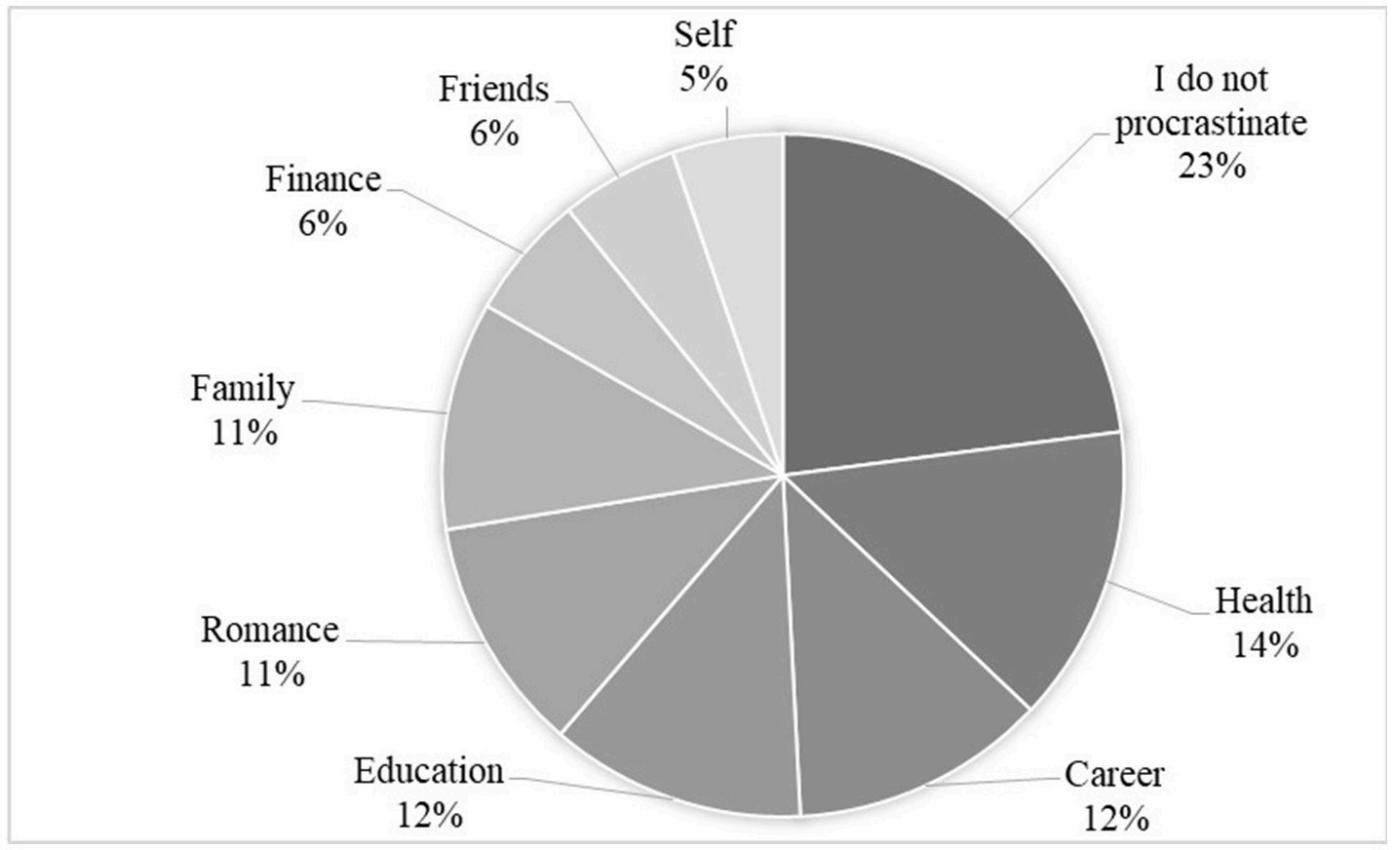
Distribution of life-domains that respondents chosen as most influencing on their lives (open-ended question).

Finally, in order to answer our last question, we asked the respondents to reflect in which life-domain their procrastination influenced them the most. We calculated the frequencies of each life-domain that was reported by participants. Our findings indicate that 23% reported that they do not procrastinate at all and, therefore, procrastination did not influence them in any life-domain (see Figure 2). Fourteen percent replied that procrastination influenced them the most in maintaining a healthy life style; 12% reported being influenced the most in education and career life-domains each, and 11% in the romantic relationships and family life-domains each; 6% reported being influenced the most in finance and friends each; finally, 5% reported the self-development life-domain as the most influenced life-domain. Interestingly, respondents reflected being mostly influenced by procrastination only in 8 out of 11 life-domains (excluding leisure time, community and parenting), and a discrepancy between the intensity and the influence of procrastination in life-domains was indicated as well. While our quantitative results showed that respondents reported high procrastination, especially in health life domain, followed by moderate procrastination in leisure time and self-development life domains, our qualitative findings suggested that respondents felt procrastination influenced them in health, education, career, and relationships similarly.

## Discussion

Following advances in the procrastination literature, and the need to better understand procrastination in a life-span perspective (Mann, 2016), the present study examined general and life-domain procrastination in highly educated adults. The main goal was to get initial clues to the generality and specificity of procrastination in highly educated adults in Israel. Following other adult procrastination studies (Klingsieck, 2013; Steel and Ferrari, 2013) we examined the general and specific presence of adult procrastination across 11 life-domains. Our main findings supported other adult procrastination studies that indicated low rates of general procrastination in highly educated adults (Beutel et al., 2016; Svartdal et al., 2016), and suggested that life-domain procrastination in adults is specific to some degree (Klingsieck, 2013). Further when examining the frequencies of respondents that highly procrastinate, we noticed that only 1% of our respondents reported high general procrastination, while 41% reported high procrastination in maintaining health behaviors and 28% in spending leisure time. These results enhance the specificity notion of procrastination in highly educated adults and also may align with Ferrari et al. (1995) argument that procrastination rates do not decrease with age, but actually reflect different forms of procrastination. Further our results indicated that highly educated adults in our sample reported significantly higher levels of procrastination in two life-domains: *Maintaining health behaviors* and *spending leisure time* and lowest procrastination in *Parenting*. These results can be interpreted from different angles. First when compared with Klingsieck's (2013) results regarding life-domain procrastination in students, we noticed that the two life domains most procrastinated by highly educated adults are different than the most procrastinated life-domains reported by students (i.e., work and academics). This might reflect either /or a developmental or a situational effect on procrastination in adults, suggesting that both maturation and context may be playing an important role in the way procrastination presents itself in highly educated adults. Further our results indicated that the two life domains mostly procrastinated by highly educated adults (health and leisure time) are to some degree associated with adult physical and psychological well-being (Ryff, 2014) and overall wellness (Stussman et al., 2015) and therefore needs to be carefully monitored and addressed. This stresses the need to further study adult procrastination in these specific life-domains (Sirois, 2015) and in other life domains that procrastination has not been studied yet, or have been studied briefly (Mann, 2016; van Eerde, 2016).

Our results further indicated that about 25% of our respondents reported high procrastination in 4 and more life-domains. This finding may to some degree reflect or support other findings that suggest the existence of chronic procrastination in about 20–25% adults in Western cultures (Harriott and Ferrari, 1996). We did not specifically measure chronic procrastination, but from interpreting the literature, it seems that people, who highly procrastinate in four and more life domains, may present a chronic pattern (Díaz-Morales et al., 2008). In addition our results show that the general tendency to procrastinate was moderately associated with three life-domains: Career, Education, and Finance, that seem to reflect the achievement oriented life-domains, and maybe suggest that the general tendency to procrastinate is a behavioral tendency that mostly reflects the ability to socially or timely perform (Steel, 2010). Therefore, in those life-domains that highly educated adults are not required to socially perform, their life-domain procrastination pattern seems to be is somewhat different.

Finally the specificity of procrastination was presented in the reflections of our respondents on the life-domains that procrastination mostly influenced their lives. Procrastination in health (14%), career and education (12%), romance and family (11%) influenced our respondents' lives more than procrastination in other life-domains. Although most of these life-domains were not reported as highly procrastinated (excluding health), yet people felt they were most influenced in these domains. This may suggest that not only that the presence of procrastination differs across life-domains but maybe more importantly, the effects and consequences of this behavior differ across life-domains. In addition it increases the need to study the presence of adult life-domain procrastination and its' effects mutually, without assuming that high procrastination has more negative consequences on the adult.

Taken together our findings, although descriptive and initial in nature, suggest that life-domain procrastination in highly educated adults is domain specific to some degree. It also suggest that adults' life-domain procrastination is reported more present in self-oriented life-domains that seem to be related to adult well-being (Ryff, 2014) and low in parenting. Our findings also show that highly educated adults feel that life-domain procrastination influences them the most in health, achievement-oriented life-domains and relationships, although frequencies in most of these domains were only moderate.

As with any study, the present study may present with potential limitations that are worth acknowledging. First, the use of self-report questionnaires may be accompanied by possible associated biases (e.g., social desirability biases, short-term recall biases, etc.). Second, we used a single-item to represent each life domain. Although some authors suggest that single-item measures may suffice (Wanous et al., 1997; Drolet and Morrison, 2001; Bergkvist and Rossiter, 2007), others argue that constructs with at least two items are considered as reliable and valid (Yong and Pearce, 2013). Therefore, future studies are advised to measure life-domain procrastination using instruments where each life-domain would be represented by several items. Third, we applied a non-probability sampling strategy for this study, resulting by several biases, such as over-representation of female population and of some professions in the sample. In order to examine the generalizability of the results, it would be helpful for future research to utilize larger random samples drawn from different populations and different countries. Fourth, while we were interested in procrastination patterns from the life-span perspective, we did not test them over time and used a cross-sectional design that does not allow examining the results in a time perspective. In order to better understand procrastination patterns and whether and how they change, future research should apply a longitudinal design. Despite these limitations, the findings of this study support the life-domain specificity of procrastination in adults. By that, they encourage the differentiation between procrastination in different life-domains in the terms of theoretical approaches, diagnostic tools, and intervention programs. Our findings clearly indicate that further research to differentiate adult procrastination's specific characteristics in various life-domains needs to be the goal of future studies.

## Ethics statement

This study was carried out in accordance with the recommendations of Tel-Hai Academic College Ethics committee with written informed consent from all subjects. All subjects gave written informed consent in accordance with the Declaration of Helsinki. The protocol was approved by the Tel-Hai Academic College Ethics committee.

## Author contributions

All authors listed have made a substantial, direct and intellectual contribution to the work, and approved it for publication.

### Conflict of interest statement

The authors declare that the research was conducted in the absence of any commercial or financial relationships that could be construed as a potential conflict of interest.

## Appendix

**Appendix 1:** Life-domain procrastination (LDP) items.

To what extent do you procrastinate in each one of the following life-domains? (from 1 = *not at all/rarely to* 5 *= very often/all the time*) Career (For example: getting a better job, asking for a wage-raise) Community (For example: volunteering and helping others) Education and studying (For example: studying for exams, procrastinating in getting an academic degree) Parenting interactions with offspring (For example: spending time with children, family vacation) Family interactions with parents/siblings (For example: having dinner with your family, calling to your parents) Financial decisions/investments (For example: paying bills, opening saving plans, paying loans) Friend interactions (For example: spending time with your friends, inviting your friends to dinner) Health, exercise, diet, illness (For example: going to a gym, starting a diet, visiting a doctor) Leisure, sports, recreation, travel (For example: going to vacation, joining a sport group) Romance, love, dating, marriage (For example: finishing a relationship, purposing a marriage, inviting somebody for a date) Self-improvement, personal growth (For example: reading a self-help book, changing an image).
